# Filaments and phenotypes: cellular roles and orphan effects associated with mutations in cytoplasmic intermediate filament proteins

**DOI:** 10.12688/f1000research.19950.1

**Published:** 2019-09-30

**Authors:** Michael W. Klymkowsky

**Affiliations:** 1 Molecular, Cellular & Developmental Biology, University of Colorado, Boulder, Boulder, CO, 80303, USA

**Keywords:** intermediate filament proteins, chaperones, stress response, phenotypes, mutation, background effects

## Abstract

Cytoplasmic intermediate filaments (IFs) surround the nucleus and are often anchored at membrane sites to form effectively transcellular networks. Mutations in IF proteins (IFps) have revealed mechanical roles in epidermis, muscle, liver, and neurons. At the same time, there have been phenotypic surprises, illustrated by the ability to generate viable and fertile mice null for a number of IFp-encoding genes, including vimentin. Yet in humans, the vimentin ( *VIM*) gene displays a high probability of intolerance to loss-of-function mutations, indicating an essential role. A number of subtle and not so subtle IF-associated phenotypes have been identified, often linked to mechanical or metabolic stresses, some of which have been found to be ameliorated by the over-expression of molecular chaperones, suggesting that such phenotypes arise from what might be termed “orphan” effects as opposed to the absence of the IF network *per se*, an idea originally suggested by Toivola *et al*. and Pekny and Lane.

## Introduction

Cytoplasmic intermediate filaments (IFs), together with actin-based microfilaments and tubulin-based microtubules, combine to form the eukaryotic cytoskeleton. (Here, I concentrate on cytoplasmic IFs and [almost] completely ignore the nuclear lamins as well as the septins associated with tight junctions.) Microtubules and microfilaments are unambiguously essential elements of eukaryotic cells. Notwithstanding claims that IFs are the “primary determinants of cell architecture and plasticity” ^1^ and play a “pivotal role in regulatory cell architecture and function” ^2^, the roles played by IFs are more enigmatic and context-specific than those of microtubules and microfilaments, specifically given the observation that for many (most) IF polypeptide (IFp)-encoding genes, mice homozygous for null mutations are viable and fertile ( Table 1). The goal of this review is to draw attention to complications in the interpretation of the phenotypes associated with null and antimorphic (dominant negative) mutations in IFp-encoding genes, a point also made by Bouameur and Magin ^3^.

**Table 1.** Null mutations in mice, BioGRID interacting polypeptides, and human pLI scores for intermediate filament subunit proteins.

Intermediate filament subunit protein	Number of interacting proteins BioGRID (unique)	Predicted versus expected loss of function (LoF)	Probability of LoF intolerance (pLI)	Mouse knockout
Vimentin (VIM)	315	2/14	0.96	Yes
Peripherin (PRPH)	28	16/15	0.0	Yes
Desmin (DES)	48	7/16.7	0.0	Yes
Synemin (SYNM)	22	21/26	0.0	Yes
Glial fibrillary acidic protein (GFAP)	103	9/13	0.0	Yes
NFL (NEFL)	68	Not reported	Not reported	Yes
NFM (NEFM)	52	5/14.5	0.04	Yes
NFH (NEFH)	26	7/14	0.0	Yes
Internexin (INA)	45	2/7.8	0.29	Yes
Syncoilin (SYNC)	50	4/10.6	0.04	Yes
Nestin (NES)	69	13/30	0.0	Yes
Nebulin (NEB)	27	79/249	0.0	Yes
Keratin 1 (Krt1)	96	2/19.7	0.97	Not found
Keratin 2 (Krt2)	84	5/16.3	0.07	Not found
Keratin 3 (Krt3)	24	10/12.3	0.0	Not found
Keratin 4 (Krt4)	26	9/21	0.0	Yes
Keratin 5 (Krt5)	86	3/14.2	0.47	Yes
Keratin 6 (Krt6)	None so far	9/13.8	0.0	Yes
Keratin 7 (Krt7)	22	12.6/13	0.0	Yes
Keratin 8 (Krt8)	80	10/14	0.0	Yes
Keratin 9 (Krt9)	72	7/14.5	0.0	Yes
Keratin 10 (Krt10)	86	6/17.3	0.02	Yes
Keratin 12 (Krt12)	4	16/16.3	0.0	Yes
Keratin 13 (Krt13)	72	6/10.9	0.0	Not found
Keratin 14 (Krt14)	65	4/11.9	0.07	Yes
Keratin 15 (Krt15)	148	16.2/20	0.0	Not found
Keratin 16 (Krt16)	57	13/12.3	0.0	Yes
Keratin 17 (Krt17)	182	10/14.5	0.0	Yes
Keratin 18 (Krt18)	126	2/12	0.62	Yes
Keratin 19 (Krt19)	72	6/13	0.0	Yes
Keratin 20 (Krt20)	30	10/14.1	0.0	Not found
LMNA	802	1/19	0.99	Yes
LMNB1	122	2/18	0.95	Yes
LMNB2	59	1/20	1.0	Yes

Null mutations in mice, BioGRID interacting polypeptides, and human pLI scores for intermediate filament subunit proteins included lamin A/C ^34, 35^, B1 and B2 type lamins ^36^, vimentin ^37^, glial fibrillary acidic protein (GFAP) ^38, 39^, desmin ^27, 28, 40, 41^, nestin ^42, 43^, the three neurofilament proteins (NEFL, NEFM, and NEFH) ^44– 50^, peripherin ^51, 52^, internexin ^53^, synemin ^54, 55^, syncoilin ^56^, Krt4 ^57^, Krt5 ^58^, Krt6 ^59^, Krt7 ^60^, Krt8 ^61– 63^, Krt9 ^64^, Krt10 ^65^, Krt12 ^66^, Krt14 ^67– 69^, Krt16 ^70^, Krt17 ^71^, Krt18 ^72^, and K19 ^73, 74^. These studies have been extended in mice missing all type I and type II keratins ^75, 76^. Interaction partner estimates are from https://thebiogrid.org (accessed July 4, 2019).

The cytoplasmic IFp genes appear to have evolved from the nuclear lamins ^4, 5^. In this light and given the viable phenotypes associated with many IFp-null mutations in the mouse (see below), it is interesting to note that cytoplasmic IFs have been lost in the arthropods, although they are present in other invertebrates ^6– 8^. In collembolans, copepods, and tardigrades, the cytoplasmic IFs that are present appear to be formed by lamin-like proteins ^9^. Lamins appear to be core components of eukaryotes ^5^.

While analyzing the positive and negative effects of selection on specific genetic loci is complex, we can assume that if a functional version of a gene is necessary for an organism’s survival or reproductive success, loss of function (LoF) alleles will be rare or absent from a population. The Exome Aggregation Consortium (ExAC) database ( http://exac.broadinstitute.org) contains a collection of exome sequences of 60,706 unrelated people, unaffected “by severe pediatric disease”. Allelic variants likely (although by no means certain) to produce a LoF effect, that is, stop codons and defects in splice junctions near the 5' start of the gene, were identified. Lek *et al*. ^10^ defined the probability of LoF alleles existing within this collection using the “probability of being loss-of-function intolerant” (pLI) metric. The process of generating the pLI metric is complex and described in detail in the associated supplement “Constraints” by Samocha *et al*., a part of Lek *et al*. ^10^. A gene’s pLI score is an estimate of whether or not LoF mutations in that gene, whether homozygous or heterozygous, are efficiently removed from the population by selection. At the extremes, a pLI score of zero indicates that the gene is likely to be non-essential in most situations whereas a score of one indicates that it is essential (that is, it results in lethality or reproductive failure). A gene would be predicted to be essential if the frequency of LoF alleles (under conditions commonly experienced in the population) was zero (or very low) compared with its predicted occurrence, based on the assumption that it appeared randomly and without significant selective implications.

Human population genome sequence data, such as the ExAC database ^11^, reveal essentially zero probability of being loss-of-function intolerant (pLI) scores for most IFp genes ( Table 1). The notable exceptions are vimentin ( *VIM*) and keratin 1 ( *KRT1*), which have pLI scores of 0.96 and 0.97, respectively, similar to that for the nuclear lamins (0.95 to 1.0), scores indicative of an essential gene whose inactivation by mutation leads to strong negative selection. In this light, species differences between mouse and human may be relevant ^12^. Other IFp genes with non-zero pLI scores are the keratins *KRT18* (pLI: 0.62) and *KRT5* (pLI: 0.47) and the neural IFp α-internexin ( *INA*) gene (pLI: 0.29). Nonetheless, it is unambiguously the case that mutations in IFp-encoding genes play a causal role in a number of human diseases ^13, 14^ ( http://www.interfil.org). An example is a dominant-acting missense mutation in *VIM* that disrupts IF formation, leading to “pulverulent cataract in a 45-year-old individual” ^15^.

My own introduction to IFs was through intracellular injection studies that revealed a lack of overt effects following the disruption of IF organization in the admittedly highly artificial context of cell culture ^16^ (similar to results reported by 17, 18). Subsequent studies reported effects on lipid synthesis and nuclear morphology in cultured cells that would normally express *VIM* ^19, 20^ but these phenotypes were not apparent in *VIM* ^–/–^ mice ^21^. Real progress was made when investigators moved from cultured cells to developing organisms. In *Xenopus*, KRT-type IFs were implicated in the mechanical process of gastrulation ^22^, an observation supported and extended by a recent study by Sonavane *et al*. ^23^. Mutations in genes encoding KRT IFps resulted in the mechanical fragility of mouse and human epidermis (reviewed in 24, 25). In muscle, the absence of the IF protein desmin (DES) or the expression of mutant DES led to structural defects in both skeletal and cardiac muscle ^26– 28^. Since then, increasingly thorough analyses have established the mechanical roles of IFs in cells and tissues ^3, 29– 31^.

## Unanswered questions

Which of the phenotypic effects associated with mutations in IFp-encoding genes are direct, that is, due to the absence of an intact IF network, and which are indirect, due to the redistribution of proteins normally associated with IFs, remains to be resolved. That IFps interact with cellular factors was indicated to us by the observation that *Xenopus* vimentin protein failed to assemble a filament network in *Xenopus* oocytes ^32^. The role of host cell factors has been further illustrated by studies in which human IFps were expressed in *Drosophila*, which has no cytoplasmic IFs of its own. In *Drosophila* S2 cells and mesenchymal tissues (the types of tissues that would normally express *VIM* in humans), human vimentin was unable to form filament networks; on the other hand, it formed cage-like filament networks around the nuclei of internal epithelial cells ^8^.

**Figure 1.**
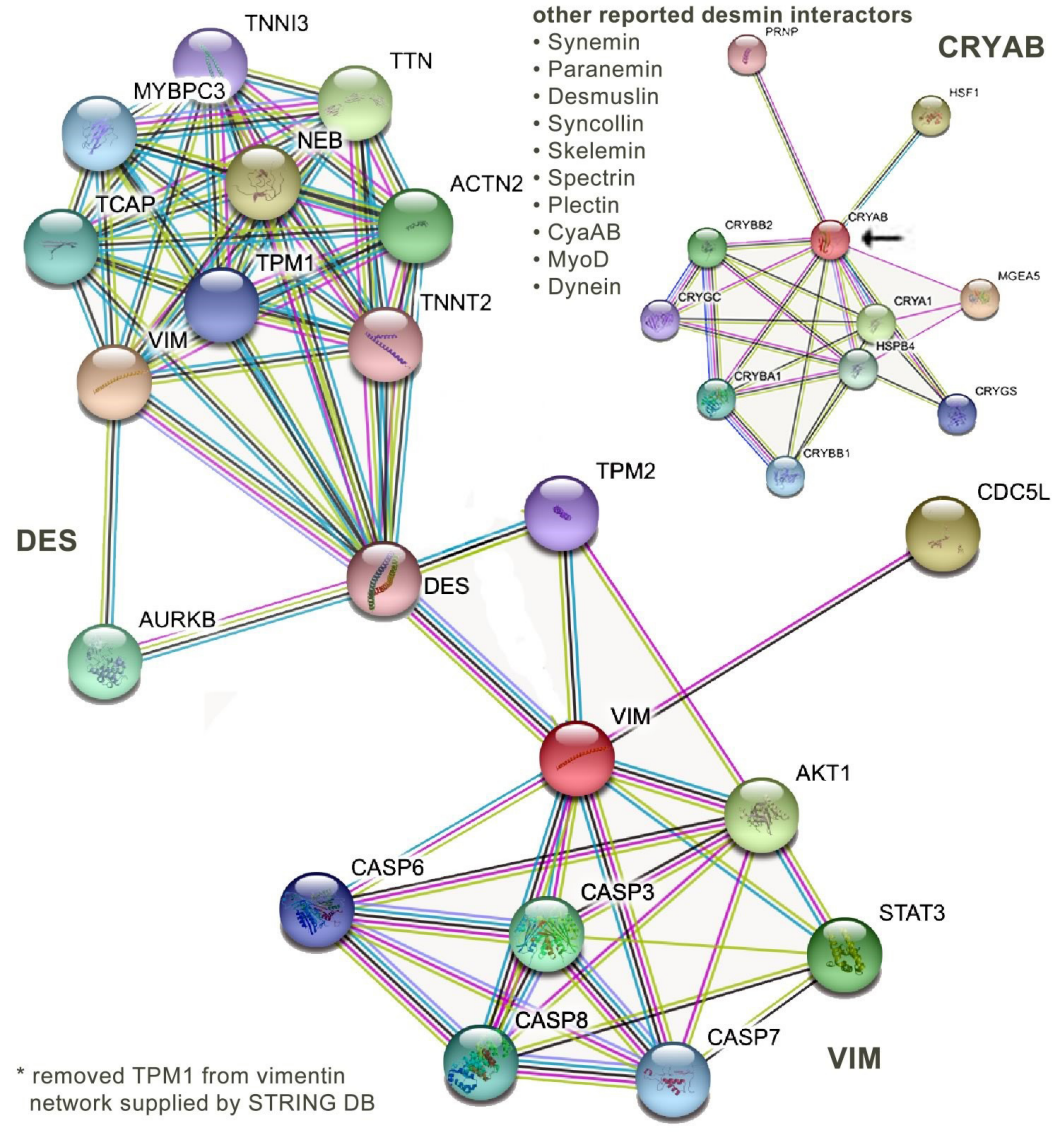
Interaction networks (derived from the STRING-DB website) for vimentin and desmin. We list the desmin-interacting proteins—from Costa *et al*. ^78^ (2004)—that are absent from either map. As an example, chaperone αB-crystallin (CRYAB) is absent. Its interaction map is displayed in the upper right hand corner.

There are a number of tools available to visualize protein–protein interaction networks ^33^. (It is worth noting the formal distinction between a polypeptide gene product and a functional protein, which may be composed of multiple different gene products and multiple subunit polypeptides. See https://bioliteracy.blog/2018/05/15/when-is-a-gene-product-a-protein-when-is-it-a-polypeptide.) An often-used tool is STRING ^77^, which displays a range of interactions graphically. Here, I have used STRING to present a crude snapshot of interactions involving VIM and DES proteins ( Figure 1). One immediately notes that a number of known DES-interacting proteins ^78^ derived from the BioGRID database ^79^ are absent ( Table 1 and Figure 1). I refer to interacting proteins that may be influenced by the absence of an IFp as orphan proteins. In the absence of an intact IF network, such orphans may adopt wayward (toxis) structures and interact inappropriately with other cellular structures, leading to secondary phenotypes, an idea originally suggested by Toivola *et al*. ^80^ and Pekny and Lane ^81^ (see also Capetanaki *et al*. ^82^). It is likely that many functionally significant interactions have yet to be identified. An example is the molecular chaperone αB-crystallin (CRYAB), whose STRING interaction network ( Figure 1) does not include any IFps. In this case, the orphan effect involves defects in the assembly of IF networks in astrocytes associated with mutations in the gene encoding glial fibrillary acidic protein (GFAP). Such mutations lead to increased levels of soluble oligomers that act to inhibit proteosome activity in Alexander disease ^83^. In mouse models of the disease, inhibition of *CRYAB* expression led to increased mortality whereas increased *CRYAB* expression “rescued animals from terminal seizures” ^83, 84^. In a sense, the chaperone provides a home or safe haven for the non-filamentous GFAP oligomers, an idea suggested by the chaperone network described by Taipale *et al*. ^85^ and others (see below).

The gigaxonin ( *GAN*) gene encodes a E3-ubiquitin adaptor protein involved in IF network organization and degradation ^86– 88^. *GAN* is mutated in the fatal human disease giant axonal neuropathy. Our studies revealed the conditional nature of the GAN-associated VIM organization phenotype in two patient-derived primary fibroblast cell lines ^89^. Of note, the GAN protein does not appear in lists of IF associated proteins or in the STRING data base. In other cell types, the absence of glial IF networks was found to lead to an increase in neuronal and glial cell division and improvements in post-trauma regeneration ^90– 92^ as well as effects on gene expression in neighboring microglia ^93^. The mechanism(s) underlying these effects have yet to be resolved.

Traub *et al*. described the interaction between a number of IFps and nucleic acids ^94– 97^. (In our own lab, we routinely purified VIM on single-stranded DNA columns.) It is worth noting that the VIM ^–/–^ mouse generated by Colucci-Guyon *et al.* ^37^ may leave the N-terminal DNA binding domain intact. Soluble (tetrameric) forms of IFps have been identified ^98^ and found in the nuclei of cells ^99^. VIM has been reported to influence transforming growth factor beta (TGFβ)-Slug (Snai2) ^100^ and nuclear factor kappa B (NF-κB) ^101^ signaling as well as the NLRP3 inflammasone ^102^, all of which are known to influence gene expression. Similarly, desmin has been reported to enter the nucleus, associated with chromatin, and influence gene expression ^103^. These observations raise the obvious question, answerable by RNA-seq (RNA-sequencing) and proteomic studies, how does the expression (or absence) of a particular IFp influence the overall pattern of gene expression? This is a question that, to my knowledge, has not been directly answered, even though VIM-free human SW13 cells and the ability to control expression of various IFps (including VIM) have been available for some time ^19, 75, 104– 108^. Steps in this direction have been made, however. These include a microarray analysis of control and Alzheimer’s disease model mice null for both *GFAP* and *VIM*; these authors reported that the expression of hundreds of genes was altered ^93^. A similar response has been found in DES ^–/–^ mice ^109, 110^. Levels of inflammation, interleukin 1 beta (IL-1β) expression, and endothelial and alveolar epithelial barrier permeability, together with tissue remodeling and fibrosis, are attenuated in the lungs of *VIM* ^–/–^ mice ^102^. The absence of KRT expression influenced epidermal barrier formation and mitochondrial lipid composition and activity in the cornified epithelia of transgenic mice ^111^. In some cases, IFp concentrations have been found to increase dramatically in the context of cell stress, suggesting that IFps themselves may act as stress proteins, part of a stress response network ^112^.

There are multiple reports of interactions between IFs and mitochondria ^111, 113– 123^, as well as with endoplasmic reticulum, which interacts with mitochondria ^124, 125^, and the microtubule-anchoring centrosome ^126^. The disruption of these interactions could lead to a range of effects, including mitochondrial dysfunction, which has been reported in a number of IFp-null mice. Given the central role of mitochondrial activity in a wide range of tissues and cellular processes ^127– 130^, such effects may be more impactful than the “primary” defects arising from the absence of the IF network itself. As an example, mitochondrial effects have been linked to the behavior of primary cilia, an organelle closely involved in a number of intra- and intercellular signaling systems active during embryonic development and within mature tissues ^131^. Abnormal mitochondrial structure, function, and activity may be involved in a wide range of IF-associated phenotypes, such as increased oxidative stress in macrophages, leading to vascular inflammation and attenuated atherosclerosis in mice ^132^, the accumulation of body fat ^133^, and differences in the growth behavior of wild-type and VIM-null cells ^115^.

Perhaps the most obvious example of IF–stress interactions and organismic phenotypes is the cardiomyopathy phenotypes observed in DES ^–/–^ mice and associated with human DES mutations ^134^. DES ^–/–^ mice display “progressive degeneration and necrosis of the myocardium” and defects in mitochondrial distribution, morphology, and function ^135, 136^. Weisleder *et al*. ^136^ observed that the most severe aspects of the DES ^–/–^ phenotype in mice were suppressed by the over-expression of Bcl2, a mitochondrial outer membrane protein involved in the regulation of apoptosis ^137^. In our own studies, expression of the related anti-apoptotic protein Bcl-xL suppressed neural crest defects associated with the loss of the transcription factor Slug (Snai2) through the activation of NF-κB signaling ^138^, suggesting the possible involvement of complex “downstream” effects. Diokmetzidou *et al*. ^139^ followed up on the rescue ability of the mouse *DES* ^–/–^ phenotype by adopting a strategy first applied by the Goldman ^83^, Messing ^84^, and Quinlan ^140, 141^ groups, who found that the expression of the molecular chaperone CRYAB ^142^ suppressed the toxicity of GFAP mutants in mouse models of Alexander disease (see above). In the case of *DES* ^–/–^-null mice, the Capetanaki group found that expression of αB-crystallin ameliorated many of the mitochondrial defects displayed in heart muscle, leading to “almost wild-type levels” of mitochondrial activity ^143^. In a related study, this group found that over-expression of tumor necrosis factor alpha (TNFα) led to expression of the simple epithelial keratins Krt8 and Krt18 in the heart; these keratins assumed many of the structural roles normally carried out by DES and rescued mitochondrial defects ^144^. In the absence of these keratins (and DES), critical desmosomal and adherens junction proteins, all known to influence intracellular signaling systems and gene expression networks, were displaced ^61, 145– 147^. These observations reinforce the idea that the loss of wild-type DES in particular, and IFps in general, can lead to the mislocalization of proteins known to play important roles in the regulation of mitochondrial function and gene expression.

Simple epithelial keratins provide a classic example of both genetic background effects and the role of IFps under conditions of cellular and tissue stress. The first reported knockout of any IFp was Krt8. In C57BL/6 mice, *Krt8* ^–/– ^animals displayed about 94% embryonic lethality ^62^. However, when crossed into the FVB/N genetic background, embryonic lethality was suppressed, although *Krt8* ^–/–^ mice displayed colonic hyperplasia and inflammatory phenotypes in desmin null mice ^148^. In 20-week-old *Krt8* ^–/– (FVB/N)^ mice, analysis of liver structure revealed no overt phenotypes associated with the absence of KRT filaments. KRT filaments do not form in this simple epithelial tissue in the absence of Krt8. On the other hand, a rapid increase in blood flow and the cellular stresses associated with partial hepatechomy led to 100% lethality in *Krt8* ^–/– (FVB/N)^ mice compared with significant levels of survival in heterozygous and wild-type mice ^149^. A similar increase in hepatechomy-associated lethality was observed in *Krt18* ^–/–^ mice ^150^ as well as in humans with KRT mutations/variants ^13, 151^. Clearly, genetic background effects, the presence of particular stresses, and cellular responses to those stresses play important roles in the various disease phenotypes associated with IFp variants ^152^.

There have been a number of reports on roles for VIM in cell migration and epithelial-mesenchymal transition (for example, 153– 157), a key developmental event associated most dramatically with the formation and migration of neural crest cells and their roles in a number of tissues, particularly the vertebrate craniofacial skeleton ^156, 158– 160^. Yet to my knowledge, no craniofacial or cell migration-dependent defects have been described in *VIM* ^–/–^ mice or *VIM* mutations/variants in humans. It remains unclear whether the phenotypes associated with aberrant *VIM* expression are due to the absence of VIM *per se* or to secondary effects involving orphaned VIM-associated proteins. An obvious experiment would be to ask whether increased expression of molecular chaperones, such as αB-crystallin, rescued any or all of such cell migratory phenotypes.

The size of the IFp gene family raises another recently identified potential complication in the link between mutation and phenotype. As reviewed by Wilkinson ( 161 and references therein), non-sense mutations can provoke a non-sense–mediated, RNA decay–based gene regulatory feedback system that can lead to the activation of (often) sequence-related genes. More generally, the viability of biological systems in the face of molecular level noise (including mutations) is enhanced by a range of adaptive molecular chaperones and feedback networks ^85, 162, 163^. Given the effects of expressing chaperones on mutant IFp phenotypes (see above), a more complete understanding of the molecular mechanisms responsible for the phenotypes associated with mutant IFp genes is likely to suggest more effective therapeutic strategies, such as the use of small molecule “chemical chaperones” ^164^, as well as a deeper understanding of the responsive interaction networks that underlie biological behaviors.
